# Prevention programmes for children of parents with a mood/anxiety disorder: Systematic review of existing programmes and meta‐analysis of their efficacy

**DOI:** 10.1111/bjc.12277

**Published:** 2021-01-06

**Authors:** Petra J. Havinga, Dominique F. Maciejewski, Catharina A. Hartman, Manon H.J. Hillegers, Robert A. Schoevers, Brenda W.J.H. Penninx

**Affiliations:** ^1^ Department of Psychiatry Interdisciplinary Center Psychopathology and Emotion regulation (ICPE) University Medical Center Groningen University of Groningen The Netherlands; ^2^ Department of Developmental Psychopathology Behavioural Science Institute Nijmegen The Netherlands; ^3^ Department of Child and Adolescent Psychology and Psychiatry Erasmus Medical Center Rotterdam The Netherlands; ^4^ Department of Psychiatry and Amsterdam Public Health Research Institute Amsterdam UMC Vrije Universiteit Amsterdam The Netherlands

**Keywords:** anxiety, bipolar, depression, offspring, prevention

## Abstract

**Objectives:**

To systematically describe the characteristics and techniques of prevention programmes for children of parents with mood/anxiety disorders. In addition, recruitment approaches and difficulties were identified and a meta‐analysis was conducted to examine the efficacy of these prevention programmes.

**Methods:**

Randomized controlled trials assessing the efficacy of a prevention programme for children (6–25 years) of parents with mood and/or anxiety disorders were included. A systematic literature search was conducted in PubMed, PsychINFO, and CENTRAL from the earliest record to March 2019. In addition, programme manuals of identified prevention programmes were requested for a content analysis.

**Results:**

Twenty‐two articles containing eight unique prevention programmes involving 1,325 subjects were identified. Programmes varied in the number and types of techniques, but all provided psychoeducation. Results suggested that recruitment via clinicians was more successful than recruitment via health maintenance organization databases. In a meta‐analysis, a significant risk difference was found in favour of prevention programmes on the risk of developing a depressive/anxiety disorder in offspring at short‐term (9–18 months follow‐up; RR = 0.37, 95% CI [0.21; 0.66]) and long‐term follow‐up (24 months or longer follow‐up; RR = 0.71, 95% CI [0.57; 0.87] and on symptom levels in offspring at post‐intervention (SMD = −0.19, 95% CI [−0.36; −0.02]) and at 12‐months follow‐up (SMD = −0.31, 95% CI [−0.57; −0.06]).

**Conclusions:**

The prevention programmes combined psychoeducational elements with skills training and/or cognitive‐behavioural therapy elements. The recruitment process and the content of these programmes are sometimes insufficiently described. Nevertheless, they appear to be effective, indicating a need to further examine how these programmes exactly work and for whom.

**Practitioner points:**

- Preventive interventions for children of parents with mood/anxiety disorders appear to be effective in preventing these disorders in offspring.
- Available preventive intervention programmes focus mostly on psychoeducation, cognitive‐behavioural therapy, and family processes.
- More effort should be made into describing preventive interventions so that they can be easily implemented by practitioners.
- Studies should further examine why and for whom preventive interventions for children of parents with mood/anxiety disorders are effective.

## Background

Mood and anxiety disorders are prevalent and disabling disorders (Steel et al., 2014; Vigo, Thornicroft, & Atun, 2016). Previous studies suggest that children whose parents suffer from these conditions are more likely to develop a mood and/or anxiety disorder (further denoted as mood/anxiety disorder) compared to children of parents without affective psychopathology (Micco et al., 2009; Rasic, Hajek, Alda, & Uher, 2014). Children of parents with mood/anxiety disorders are thus an important target group to be addressed by preventive efforts.

Over the past decades, several prevention programmes have been designed aiming to prevent the development of mood/anxiety episodes in those children. Earlier meta‐analyses have found that prevention programmes in children of parents with mental disorders in general (Siegenthaler, Munder, & Egger, 2012; Thanhäuser et al., 2017) and depression in particular (Loechner et al., 2018) are effective in preventing mental disorders. Siegenthaler et al. (2012) included 13 unique trials that focused on children of parents with mental disorders, including depression, anxiety, alcohol dependence, and drug dependence. Prevention programmes included were family‐based, parent‐based, couple‐based, and youth‐based programmes. Meta‐analytical results showed that incidence of mental disorders in children was significantly decreased by 40% in children in intervention groups (RR = 0.60). Additional to incidence, there was an effect on internalizing symptoms (SMD = −0.22, *p* = .003), but not on externalizing symptom severity (SMD = −0.16, *p* = .12) at post‐test. Effects were not calculated for the follow‐up period. Similarly, Thanhäuser et al. (2017) focused on preventive interventions for children of parents with mental illness in general, namely substance‐use disorders, depression, anxiety, and/or eating disorders. Interventions included were family‐focused and parent‐based programmes as well as child‐focused programmes. Many of those programmes used CBT techniques. Analyses were conducted on continuous outcomes and results showed small, but significant effects on internalizing symptoms at post‐test (SMD = 0.17, *p* = .01; 17 studies) and medium effect sizes at 12‐month follow‐up (SMD = 0.45, *p* < .001; 9 studies). Similarly to Siegenthaler et al. (2012), effects for externalizing symptoms were not significant at post‐test (SMD = 0.10, *p* = .13; 10 studies). However, effects were significant at 12‐month follow‐up with small effect sizes (SMD = 0.17, *p* < .001; 9 studies). The results of these two meta‐analyses indicate that interventions for children of parents with mental illnesses can be effective. However, both studies did not differentiate between different parental illnesses and thus it is unclear whether effects apply specifically to children of parents with mood and anxiety disorders or more broadly to mental disorders in general. Lastly, a meta‐analysis by Loechner et al. (2018) focused on parental depression specifically. The study included seven unique trials focusing on family‐based, parent‐based, and adolescent‐based programmes. Results showed that effects were significant for depression incidence in short‐term follow‐up (combining assessment points that were most comparable in studies with different follow‐up periods, namely 6‐month to 15‐month follow‐up; RR = 0.56). Additionally, effects for depressive and internalizing symptoms were small, but significant at post‐intervention (*g*′ = −0.20, *p* = .005). However, effects were not sustained at short‐term and long‐term follow‐up (*p* > .12). The results of Loechner et al. (2018) are overall in line with the two previous described meta‐analyses, which focused on parental disorders as a whole. All meta‐analyses show relatively small, but significant protective effects for child psychopathology symptoms. One difference is that effects were not sustained at follow‐up in the study of Loechner et al. (2018), whereas they were for Thanhäuser et al. (2017). The paper by Siegenthaler et al. (2012) did not address longer term effects for psychopathological symptom severity.

However, previous meta‐analyses solely focused on determining the efficacy of prevention programmes. While this is not necessarily a limitation, it is unfortunate, because for implementation into clinical practice as well as for replication of randomized controlled trials (RCTs), intervention descriptions of sufficient detail are essential (Hoffmann et al., 2014). Meta‐analyses alone can give information about the effectiveness of interventions, so that researchers as well as clinicians know which interventions work and which to focus on in future trials and clinical practice. Although it is not a requirement to include information about treatment components and other detailed trial information for meta‐analyses, this information could complement meta‐analytical results and put them into perspective. Incomplete description of interventions has been a concern in the field of health sciences (Glasziou, Meats, Heneghan, & Shepperd, 2008).Clinicians are encouraged to use systematic reviews to inform their practice; however, when intervention descriptions are insufficient, translation into practice can be hampered. One way to deal with that issue is to use a newly developed checklist, called the Template of Intervention Description and Replication (TIDieR; Hoffmann et al., 2014). In this template, researchers give information about for instance the goal, procedure, materials and provider of the intervention. Moreover, it is also informative to analyse the techniques of different prevention programmes (e.g. cognitive‐behavioural components and improvement of parenting). Clinicians need to be aware what components effective prevention programmes use to prevent the onset of disorders in children of parents with mood/anxiety disorders. Additionally, such information is important to inform future trials.

A second important issue when evaluating the potential of prevention programmes is their success in recruitment. Previous investigators have pointed to the difficulties encountered by researchers and practitioners when inviting children of parents with a mental illness and their families to prevention programmes (Festen et al., 2014; Van Doesum et al., 2016). For example, professionals report they lack accurate knowledge about parental mental illness and on how to discuss parenting issues with patients (Van Doesum et al., 2016). Parents may experience stigma or do not realize the importance of intervention (Festen et al., 2014; Van Doesum et al., 2016) and children themselves may refuse to participate, for example because they do not want to become involved in parental issues. Knowledge on recruitment approaches and difficulties experienced can be used to optimize recruitment strategies and is of high importance to take informed decisions on whether or not to start a trial or to implement a prevention programme.

In order to extend previous meta‐analyses which have mainly focused on the efficacy of prevention programmes, the main aim of the present review is to systematically describe the characteristics and techniques of prevention programmes for children of parents with mood/anxiety disorders. In addition, this paper aims to identify recruitment strategies and difficulties. Moreover, for completeness, we also evaluated the efficacy of these programmes in terms of their ability to prevent the onset of mood/anxiety disorders and to reduce mood/anxiety symptoms, as previous meta‐analyses were either focused on mental illnesses as a whole (Siegenthaler et al., 2012; Thanhäuser et al., 2017) or solely on depression, but not anxiety (Loechner et al., 2018). Due to the high comorbidity rates found for mood and anxiety disorders (Lamers et al., 2011), we included prevention programmes for children of parents with mood and/or anxiety disorders. The reviews by Siegenthaler et al. (2012) and Thanhäuser et al., (2017) did not differentiate between different disorders. Thus, from these reviews it is not clear whether effects are specific to mood/anxiety disorders or mental illness as a transdiagnostic factor. While Loechner et al. (2018) took a disorder‐specific approach by focusing only on parental depression, the study was focused on unipolar depression. In contrast, we focused on mood disorders as a whole (including unipolar and bipolar depression) and also included trials with parents with anxiety disorders, due to their high comorbidity. Moreover, as will be discussed later, we included four additional studies that were not included by the review from Loechner et al. (2018). However, we would like to note that given these recent reviews, the meta‐analysis was a rather secondary goal. The unique contribution of the present paper is the content analysis of characteristics and techniques of prevention programmes as well as the analysis of the recruitment strategies. Both information that have been lacking in previous meta‐analysis that solely focused on the effectiveness and not on the content of the intervention and trials.

## Method

### Search strategy and selection criteria

Results were reported according to the PRISMA checklist. A literature search was conducted in PubMed, PsychINFO, and Cochrane Central Register of Controlled Trials (CENTRAL) from the earliest record to March 2019. Keywords encompassed (1) children of parents with mood/anxiety disorders, (2) preventive interventions, and (3) randomized controlled trials (RCT; see Appendix S1 for search strings). In addition, reference sections of identified papers and reviews were screened for additional studies.

To be included in the present study, a study was required to: (1) examine children aged 6–25 years who have a parent with a mood (depression or bipolar disorder) and/or anxiety disorder (i.e. projects with mental illnesses in general were not considered), (2) assess the efficacy of a prevention programme on the onset of a mood/anxiety disorder and/or mood/anxiety symptom outcome in the child, (3) be an RCT, and (4) be written in English, German or Dutch. Studies on pharmacological interventions and studies including offspring who already met diagnostic criteria for a mood/anxiety disorder were excluded. Two researchers independently screened all identified abstracts and then compared their results to resolve disagreements.

### Data extraction and data analysis

All data extraction was done in duplicate by two authors (PH and DM), and discrepancies were resolved by discussion. We first extracted information about the general characteristics of the studies (e.g. name of the intervention and target group). In order to systematically identify characteristics of the included prevention programmes, the recently introduced TIDieR (Template for Intervention Description and Replication) checklist (Hoffmann et al., 2014) was used, which aims at improving reporting of intervention details in systematic reviews. In order to give a more detailed overview about the content of the prevention programmes, we additionally extracted data on the techniques used. The data extraction template contained information on whether psychoeducation, skills training, and CBT techniques were used and whether strengthening social support was addressed in the prevention programme. For the analyses on the content of prevention programmes, we requested programme manuals. In all but one case (Compas et al., 2009), intervention manuals were received. For that study, information was based on published articles.

Additionally, we extracted recruitment approaches, percentage of contacted participants that actually participated in the trial, percentage of participants actively refusing to participate, time needed to recruit the participants, and explicit statements regarding recruitment problems.

Lastly, we examined the efficacy of the preventive interventions by conducting a meta‐analysis. The presence of a mood/anxiety diagnosis in offspring during follow‐up was our primary outcome. We clustered data of several time points into short‐term follow‐up (i.e. 9–18 months) and long‐term follow‐up (i.e. 24 months or longer). Secondary outcomes were mood (depressive and bipolar) or anxiety symptom severity in offspring at post‐intervention and at 12‐months follow‐up. These time‐frames were chosen to maximize harmonization between the different studies. If multiple informants provided information on offspring’s symptom levels, interviewer ratings were preferred over self‐report ratings and self‐report ratings over parent ratings. Thus, although disorder onset was our primary outcome, we also included studies that only reported on symptom severity. To summarize the effect of trials, the risk ratio (RR) was calculated for dichotomous outcomes and standardized mean difference for continuous outcomes (SMD; Hedges’ *g*). Dichotomous effects were weighted using the Mantel–Haenszel method and continuous effects were weighted by the inverse of variance. Heterogeneity was assessed using the *I* ^2^ statistic (Higgins & Thompson, 2002). Statistical analyses were performed in RevMan 5.3. Publication bias was examined by visual inspection of funnel plots. We assessed risk of bias following the guidelines of the Cochrane Handbook for Systematic Reviews of Interventions.

## Results

### General characteristics of included studies

**Figure 1:**
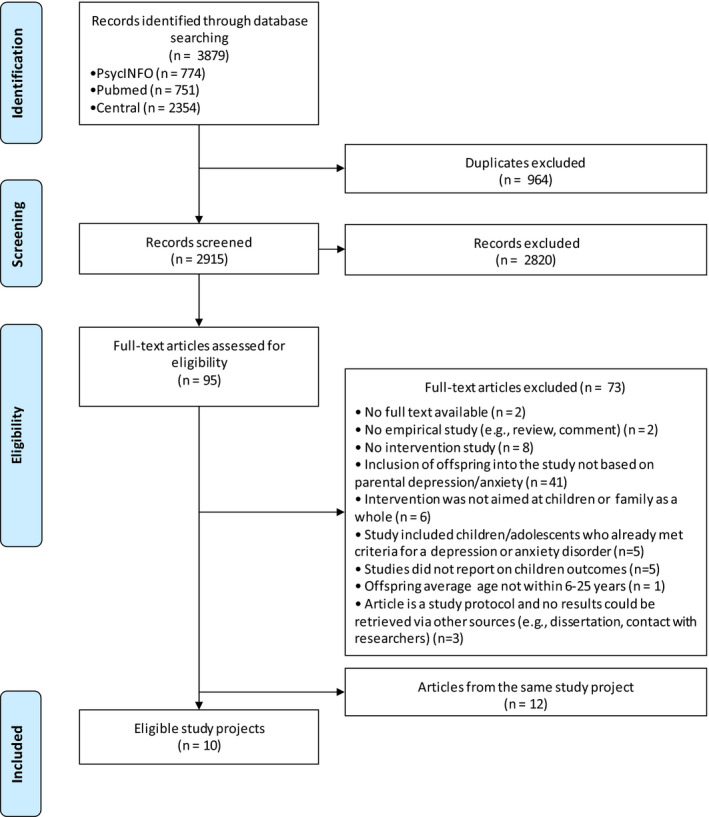
Flow‐chart.

A total of 22 articles reporting on ten study projects were included (Figure 1 and Table 1). Characteristics of the included studies are presented in Table 1. The ten study projects included a total of 1,325 subjects with a sample size ranging from 30 to 316 participants (mean = 133). The percentage of female offspring ranged from 43% to 100% (mean = 57%), and mean age ranged from 8.7 to 14.8 years (mean = 12.3). The type of control condition varied. Five studies compared a prevention programme with an informational control condition, three with a waiting list control condition, and two with care‐as‐usual. The follow‐up duration ranged from 3 to 75 months with four studies reporting a follow‐up duration of 24 months or more. Attrition ranged from 7% to 29% (mean = 15%).

**Table 1:** General characteristics of included studies

Study reference	Name of the intervention	Target group	*N* allocated (int/con)	Mean age children	% female	Assessment parental disorder	Control group	Follow‐up in months	Attrition
Beardslee et al. (1997)^a^ Beardslee, Gladstone, Wright, and Cooper (2003) Beardslee, Wright, Gladstone, and Forbes (2007)	Hope, Meaning, and Continuity	Parents with mood disorder and their children aged 8–15 years	138 (78/60)	11.6	43	SADS‐L	Informational control condition (lecture intervention in group format)	Post, 12, 24, 36, 48	17%
Clarke et al. (2001)	Coping with Stress Course	Adolescent offspring aged 13–18 years of parents with MDD and/or dysthymia Additional inclusion criteria: Current subsyndromal depressive symptoms in offspring	94 (45/49)	14.6	64	F‐SADS	Care as usual	Post, 12, 24	21%
Compas et al. (2009)^a^ Compas et al. (2010) Compas et al. (2011) Compas et al. (2015) Bettis et al. (2018)	Family group cognitive‐intervention	Parents with MDD and their children aged 9–15 years	242 (121/121)	11.5	50	SCID	Informational control condition (written information)	Post, 6, 12, 18, 24	12%
Garber et al. (2009)^a^ Beardslee et al. (2013) Brent et al. (2015) Garber et al. (2018)	Coping with Stress Course – Revision	Adolescent offspring aged 13–17 years of parents with MDD and/or dysthymia Additional inclusion criteria: Current subsyndromal depressive symptoms in offspring and/or History of depressive disorder (at least two months in remission) in offspring	316 (159/157)	14.8	59	SCID	Care as usual	Post, 3, 9, 21, 33, 75	12%
Ginsburg (2009)	Coping and Promoting Strengths	Parents with anxiety disorder and their children aged 7–12 years	40 (20/20)	8.9	45	ADIS	Waitlist	Post, 6, 12	18%
Ginsburg et al. (2015)^a^ Pella, Drake, Tein, and Ginsburg (2017)	Coping and Promoting Strengths	Parents with anxiety disorder and their children aged 6–13 years	136 (70/66)	8.7	56	ADIS	Informational control condition (Written information)	Post, 6, 12	13%
Goldstein et al. (2018)	Interpersonal and Social Rhythm Therapy	Adolescent offspring aged 12–18 years of parents with bipolar disorder	42 (21/21)	14.1	50	Medical records and SCID	Data‐informed referral (45 min face‐to‐face contact with parents and children)	1.5, 3, 4.5, 6	10%
Mason, Haggerty, Fleming, and Casey‐Goldstein (2012)	Project Hope	Parents with elevated levels of depressive symptoms and their children aged 12–15 years	30 (16/14)	13.9	44	QIDS‐SR	Waitlist	Post, 5	7%
Rasing, Creemers, Janssens, and Scholte (2013) Rasing et al. (2018)^a^	Een Sprong vooruit (A jump forward)	Adolescents aged 11–14 years with perceived parental anxiety/depression Additional inclusion criteria: Elevated depressive or anxiety symptoms in offspring	142 (69/73)	12.9	100	Self‐report via adolescent	Waitlist	Post, 6, 12	8%
Solantaus et al. (2010)^a^ Punamaki et al. (2013)	Hope, Meaning, and Continuity	Parents with mood disorder and their children aged 8–16 years	145 (67/78)	N/A	N/A	Medical records	Informational control condition (Let's Talk about the Children, discussion with parents to assess child’s situation and how to support)	Post, 4, 10, 18	29%

Note

ADIS = Anxiety Disorders Interview Schedule for DSM–IV; F‐SADS = Family Schedule for Affective Disorders and Schizophrenia; QIDS‐SR = Quick Inventory of Depressive Symptoms‐Self Report; SADS‐L = Schedule for Affective Disorders and Schizophrenia‐Lifetime Version; SCID = Structured Clinical Interview for DSM.

^a^ Main study reference.

### Characteristics of prevention programmes

Of the ten studies, two reported on the same prevention programme (Coping and Promoting Strengths programme; Hope, Meaning and Continuity) resulting in eight unique prevention programmes to be included in our content analysis. In Table 2, these programmes are described according to the TIDieR checklist (Hoffmann et al., 2014). Studies that were included focused on children of parents with the following disorders: parents with depressive disorders (five programmes), parents with bipolar disorder (one programme), parents with anxiety disorders (one programme), and parents with anxiety or depressive disorder (one programme). The latter programme concerned a transdiagnostic programme, targeting both symptoms of depression and anxiety. Four programmes were characterized as family‐focused and four targeted offspring in particular. All programmes were conducted face‐to‐face. The number of sessions varied from 6 to 15, and four programmes provided booster sessions. Moreover, all programmes reported high levels of programme fidelity. Control conditions were also described according to the TIDieR (Appendix S2).

**Table 2:** TIDieR checklist for included prevention programmes

Name of intervention	Why	What (materials)	What (procedures)	Who provided	How
Coping with Stress Course Clarke (2001)	Intervention focuses on training cognitive‐restructuring skills and techniques for modifying irrational or negative self‐statements and thoughts to better cope with stress. By modifying these irrational or negative self‐statements and thoughts, the interventions aims at preventing depression	Teen workbook, index cards, group discussions, role‐play, group activities, balloons	In the teen workbook, there are a number of exercises for the adolescents to identify, challenge and change irrational or negative thought. Examples include Comics, which are used to learn to the ABC technique and to develop positive counter thoughts. The mood diary is another example, in which adolescents learn to identify their negative feelings and the events/thoughts that are associated with these. Index cards are used to record negative thoughts. Group discussions are used to discuss learned material (e.g. negative thoughts: What are negative thoughts, what are some ways to deal with activation evens; coming up with a consensus regarding which approach is best). Group activities are used to on the one hand complete exercises (e.g. list all possible causes of depression) and on the other hand for adolescents to share one of their favourite hobbies. Balloons used as a method to get rid of negative thoughts	Therapist with a master's degree that was trained in the approach	Face‐to‐face treatment in group of adolescents
Coping with Stress Course (revision) Garber (2009)	Intervention focuses on training cognitive‐restructuring skills and techniques for modifying irrational or negative self‐statements and thoughts to better cope with stress. By modifying these irrational or negative self‐statements and thoughts, the interventions aims at preventing depression. In addition, learning problem solving skills, behavioural activation, relaxation, and assertiveness is also thought to decrease the risk of developing a depressive disorder	Teen workbook, index cards, group discussions, role‐play, group activities, balloons, 6 helpful questions	In the teen workbook, there are a number of exercises for the adolescents to identify, challenge and change irrational or negative thought. Examples include Comics, which are used to learn to the ABC technique and to develop positive counterthoughts. The mood diary is another example, in which adolescents learn to identify their negative feelings and the events/thoughts that are associated with these. Index cards are used to record negative thoughts. Group discussions are used to discuss learned material (e.g. negative thoughts: What are negative thoughts, what are some ways to deal with activation evens; coming up with a consensus regarding which approach is best). Group activities are used to on the one hand complete exercises (e.g. list all possible causes of depression, learn mindfulness techniques) and on the other hand for adolescents to share one of their favourite hobbies. Balloons used as a method to get rid of negative thoughts. 6 helpful questions are questions that help to learn how to best come up with positive counter thoughts	Therapists who were at least masters‐level clinicians trained and supervised by a Ph.D.	Face‐to‐face treatment in group of adolescents
Coping and Promoting Strengths Ginsburg (2009) Ginsburg (2015)	Intervention focuses on increasing children’s strength and resilience by teaching specific skills (e.g. cognitive and behavioural coping, problem‐solving) on reducing known risk factors associated with the onset and maintenance of anxiety in children (e.g. distorted thinking, avoidant behaviour, parental overprotection, family conflict) and on increasing knowledge of anxiety and its disorders in order to improve communication among family members, instil hope for positive outcomes, and help child/family make sense of illness	Family folder with handouts, diaries, relaxation tapes/CDs, discussions, role‐play, fear hierarchy	Family folder with handouts (e.g. Anxiety Facts, Protective Factors, Anxiety Signs & CBT, Skills List, Parenting Tips) are used to provide information and tips for parents and children, so that they can review them. Diaries (e.g. Parent SLIPS to monitor parenting strategies) are used to monitor and keep track of emotions and behaviours and make connections between thoughts, behaviours and feelings. Relaxation Tapes/CDs are used so that families can practice relaxation techniques that they learned during the session at home. Discussions are used for instance to practice material or get to know more information about the effect of parental anxiety on family. Role‐play is used to modify parental behaviours towards the child. Fear hierarchy is used to make a list of anxious objects/situations for the family and to select exposures and rewards for these different objects/situations, starting with the easiest one	Trained therapists (qualifications not further specified)	Face‐to‐face treatment with individual families
Even spring vooruit (A jump forward) Rasing (2017)	The programme aims to prevent depression and anxiety by using techniques based on cognitive‐behavioural therapy, behavioural activation, and exposure	Adolescent workbook, group exercises, inbox cards	In the workbook, there are a number of exercises based on CBT for the adolescents to identify, challenge and change irrational or negative thoughts. Group exercises, discussions, and homework are used to practice the material. On the inbox cards, adolescents are asked to describe situations they feel sad about or angry	At least psychologists at master level	Face‐to‐face treatment in group of adolescents
Family group cognitive‐behavioural intervention Compas (2009)	The main focus on this programme is to educate families about depressive disorders, increase family awareness of the impact of stress and depression on functioning, help families recognize and monitor stress, facilitate the development of adaptive coping responses to stress, and improve parenting skills	Family meetings, videotapes, role‐play	During the sessions, skills are taught through didactic instruction, viewing a videotape, modelling, role‐playing, and homework assignments. Parents learn parenting skills (i.e. praise, positive time with children, encouragement of child use of coping skills, structure, and consequences for positive and problematic child behaviour) from one facilitator, and children learn skills for coping with their parent’s depression from the other facilitator	Social workers and doctoral students	Face‐to‐face treatment with group of families
Hope, Meaning, and Continuity Beardslee (1997) Solantaus (2010)	The central goals of this intervention are to facilitate family discussion of parental affective illness and its impact on the family and to help parents identify and foster healthy coping strategies in their children	Family meetings and discussion, psychoeducational written materials for families	Family meetings to develop a shared narrative of family depression, which helps children to better understand their parental illness and its effect on the family. Written psychoeducational material helps families to develop the questioning spirit and seek out materials on their own is an important part of the best way to cope with this illness	Licensed social workers or clinical psychologists who were rigorously trained in the intervention strategies	Face‐to‐face treatment with individual families
Interpersonal and Social Rhythm Therapy Goldstein (2018)	The intervention includes: (1) psychoeducation about risk for BP; (2) Social Rhythm Therapy (SRT) aiming to establish and maintain stable routines to protect against onset of mood symptoms in vulnerable individuals; and (3) Interpersonal psychotherapy (IPT) centring on the adolescent's feelings about being offspring of parents with bipolar disorder, and linking stressful family events to mood	Handouts (e.g. closeness circle, my family tree), family meetings, social rhythm metric	Handouts are tools in the psychoeducation process, for example of information about symptoms of bipolar or identifying persons close to the adolescents. Family meetings are there for psychoeducation about bipolar disorder in adults and the associated risk for adolescents Social rhythm metric is used to develop a more regular routine of sleep and daily activities in order to help ‘set’ (or ‘bolster’) the circadian system	Experienced therapists (3 Master's level Licensed Clinical Social Workers, 1 Doctoral level Clinical Psychologist)	Face‐to‐face treatment with individual adolescents
Project Hope Mason (2012)	The main focus of the programme is on helping to strengthen communication and positive relationships in these families and to teach specific skills (e.g. problem‐solving) in order to help the adolescents avoid developing depression, drug abuse and other serious problems	Workbook (examples from the workbook include handouts or social support network map), role‐play and practice situations, family meetings, family activities	In the workbook, there are a number of handouts, that provide information and tips (e.g. handout about adolescent development or handout to work together as a family to prevent the adolescent from getting depressed or using drugs) Social support network maps are used for obtaining, structuring and feeding‐back information on informal and/or formal components of the adolescent's support network. Role play and practice situations are used to apply knowledge, for instance parents participate in practice situations to apply learned communication skills. Family activities are given as homework and used to enhance family cohesion	Trained masters‐level clinicians with backgrounds in family intervention	Face‐to‐face treatment with individual families

Name of intervention	Where	When and how much	Tailoring	Modifications	How well (planned)	How well (actual)
Coping with Stress Course Clarke (2001)	HMO clinic offices	15 sessions lasting 1 hr Conducted 2–4 times per week No booster sessions 3 informational meetings for parents	Leaders are welcome to modify the lectures, examples, and vary the exercises at their own discretion as they become more comfortable with the various content areas, but the major points made in the narrative should be retained	Not described	All sessions were digitally audio recorded. In addition, therapists receive ongoing supervision	All intervention sessions were audiotaped and 2 or 3 sessions were randomly selected form each group and rated by a senior supervisor on a 10‐item fidelity scale to assess therapist adherence to the study protocol. Mean therapist compliance was 95.9% (*SD* 3.9%; range 90–100%) across 15 rated sessions
Coping with Stress Course (revision) Garber (2009)	Not clear	8 sessions lasting 90 min Conducted weekly 6 monthly booster sessions 2 informational meetings for parents	Leaders are welcome to modify the lectures, examples, and vary the exercises at their own discretion as they become more comfortable with the various content areas, but the major points made in the narrative should be retained	Not described	All sessions were digitally audio recorded. In addition, therapists receive ongoing supervision	An early and a late session were randomly selected from each group (total of 12.5% of all sessions; *n* = 18) and rated by a senior supervisor using a 9‐item fidelity scale. Therapist compliance rating scores ranged from 88.1% to 95.8%
Coping and Promoting Strengths Ginsburg (2009) Ginsburg (2015)	Generally in therapist office	8 sessions lasting 1 hr Conducted weekly 3 monthly booster sessions	Not described	Not described	Ginsburg (2009): A detailed session‐by‐session intervention manual with session handouts, weekly supervision, and weekly progress notes documenting the content of each session that the first author reviewed weekly were used to enhance adherence. Ginsburg et al. (2015): Independent evaluators rated adherence to specific session objectives (e.g. explaining the intervention model of anxiety reduction, teaching relaxation or cognitive restructuring techniques)	Ginsburg (2009): Adherence was not formally evaluated. Ginsburg et al. (2015): The average adherence ratings per family ranged from 86.36% to 100%, and the mean adherence rating across all sessions was 97.58% (SD = 3.51), reflecting high levels of clinician adherence
Een sproong vooruit (A jump forward) Rasing (2017)	Not described	6 sessions lasting 90 min Conducted weekly No booster sessions	Not described	Not described	Treatment integrity was determined by assessing the percentage of the total programme that was actually delivered, that is, how many instructions and exercises the programme were actually given to and done by the participants	The prevention programme was delivered with integrity in all groups (*M* = 95%, *SD* = 2.47; range 91–98%)
Family group cognitive‐behavioural intervention Compas (2009)	University offices	8 sessions (duration not described) Conducted weekly 4 monthly booster sessions	Not described	Not described	A detailed list of the content of each group intervention session was developed from the manual. Five individuals not involved in delivery of the intervention were trained to code for presence vs. absence of each content area or strategy of the intervention for each session. Intervention sessions were audio recorded, and 20% were randomly selected for fidelity coding	The ratio of the number of checklist items covered during the sessions relative to the number of items that should have been covered was 92%. Reliability across coders was calculated for 31% of the sessions that were coded and yielded 93% agreement
Hope, Meaning, and Continuity Beardslee (1997) Solantaus (2010)	Family’s home or clinician’s office	6–10 sessions lasting depending on family’s needs Conducted weekly 1 booster session after 6 months	Authors provide a set of guidelines and principles that can be adapted flexibly to different settings while the core ideas remain	Not described	Beardslee et al. (1997): To ensure fidelity to the clinician‐facilitated protocol, a detailed rating of key sessions (i.e. the meeting with the child[ren], planning for the family meeting, the family meeting) was conducted. Before rating any transcripts, an adherence standard of 80% was set. Clinicians attended weekly meeting for supervision. Solantaus et al. (2010): The fidelity of the interventions was ascertained by logbooks filled out by practitioners. For the FTI (control condition), the logbooks listed the manualized topics for discussion and the practitioners marked down choices ‘Discussed, yes/no’	Beardslee et al. (1997): The clinical interventions of 10 families were rated (37 sessions), and an overall score was obtained by summing the three ratings. Raters for fidelity were not project clinicians and had no knowledge of the families’ treatment. Overall, interrater reliability with the scales was excellent (intraclass correlations ranged from .89 to .99), and adherence to the protocol was similarly stellar (86.4% for the family meeting, 91.7% for the child session). Difference in adherence among the four clinicians was non‐significant. Solantaus et al. (2010): The logbooks filled out by clinicians showed that both interventions were carried out with fidelity. All interventions included all of the different session types. No specific information on the degree of fidelity
Interpersonal and Social Rhythm Therapy Goldstein (2018)	Not described	8 in‐person sessions Conducted over 6 months No booster sessions	For each family, the information will need to be adapted based on (1) the adolescent’s family history; (2) the adolescent’s mental health history and current presentation; (3) the family’s current level of knowledge about bipolar disorder; (4) the adolescent’s age, developmental level, and insight/experience/exposure to the afflicted family member’s illness; (5) the family’s level of interest in psychoeducational material related to bipolar disorder	Not described	All staff attended training with the first and senior authors that included manual review, role plays, and discussion of videotaped pilot IPSRT sessions. Therapists participated in weekly group supervision involving videotape review and discussion of session content	A doctoral‐level rater trained and maintained at an acceptable level of IRR (ICC ≥ 0.8) with the senior author rated a random sample of 10 sessions from each study therapist using a modified version of the 22‐item IPSRT Therapy Rating Scale (Wagner, Frank, & Steiner, 1992). Mean total scores indicated high fidelity to the model among all therapists for each of the three treatment phases (1–5 scale where 1 = no IPSRT focus and 5 = extensive IPSRT focus; mean score initial phase = 4.3, intermediate phase = 3.9, final phase = 3.9)
Project Hope Mason, 2012	Family's home	10 sessions lasting 60–90 min Conducted weekly No booster sessions	Intervention specialists are encouraged to follow the curriculum protocol closely, and at the same time remain responsive to the family’s individual situation and needs. They are welcome to change the wording at their own discretion.	Not described	Treatment integrity was determined by assessing the percentage of the total programme that was actually delivered, that is, how many instructions and exercises the programme were actually given to and done by the participants	The prevention programme was delivered with integrity in all groups (M = 95%, SD = 2.47; range 91% ‐ 98%)

### Techniques used in prevention programmes

Table 3 shows that the prevention programmes varied in the number and types of techniques used. All programmes provided psychoeducation on the aetiology and symptoms of mood/anxiety disorders as well as on how parental mental illness impacts the family. For instance, in the Hope Meaning and Continuity programme, the clinician discusses symptoms of mood disorders and examines the experiences families that reflect the parental depression together with parents and children. In addition, as in the Coping with Stress Course (revision), offspring learned that despite their familial risk they are not ‘doomed’ to develop a mental illness if they strengthen their resilience. Additionally, in the Interpersonal and Social Rhythm Therapy, adolescents learned about the importance of establishing and maintaining stable routines for the prevention of bipolar disorder.

**Table 3:** Techniques of prevention programmes

	Psychoeducation		Skill training				Cognitive‐behavioural therapy elements			
	General knowledge about anxiety/depression	Impact of anxiety/depression on the family	Family communication	Parenting skills	Problem solving	Relaxation	Exposure	Behavioural activation	Cognitive restructuring	Strengthening social support
Programmes solely focused on adolescents										
Coping with Stress Course	Yes	Yes	N/NR	N/NR	N/NR	N/NR	N/NR	N/NR	Yes	N/NR
Clarke (2001)										
Coping with Stress Course (revision)	Yes	Yes	N/NR	N/NR	Yes	Yes	N/NR	Yes	Yes	N/NR
Garber (2009)										
Een sprong vooruit (A jump forward)	Yes	Yes	N/NR	N/NR	N/NR	N/NR	Yes	Yes	Yes	Yes
Rasing (2017)										
Interpersonal and Social Rhythm Therapy	Yes	Yes	N/NR	N/NR	N/NR	N/NR	N/NR	N/NR	N/NR	Yes
Goldstein (2018)										
Programmes focused on families as a whole										
Coping and Promoting Strengths	Yes	Yes	Yes	Yes	Yes	Yes	Yes	Yes	Yes	Yes
Ginsburg (2009, 2015)										
Family group cognitive‐behavioural intervention	Yes	Yes	N/NR	Yes	Yes	N/NR	N/NR	N/NR	Yes	N/NR
Compas (2009)										
Hope, Meaning, and Continuity	Yes	Yes	Yes	Yes	N/NR	N/NR	N/NR	N/NR	N/NR	N/NR
Beardslee (1997) and Solantaus (2010)										
Project Hope	Yes	Yes	Yes	N/NR	Yes	N/NR	N/NR	N/NR	N/NR	Yes
Mason (2012)										

Note

N/NR = No or not reported.

Another technique that was used in five of eight prevention programmes were cognitive restructuring techniques. In the Coping with Stress Course, for example, adolescents learned to recognize and deal with irrational or negative thoughts. Moreover, four prevention programmes employed techniques that addressed children’s problem‐solving skills, for instance distraction, acceptance, and help‐seeking.

Improving family communication and/or parenting skills were important components of family‐focused programmes. Family communication was targeted in three programmes, for instance by teaching the family new skills to foster communication between family members. In three programmes, parents learned how to improve parenting skills such as how to foster healthy coping strategies in children. Another component that was addressed in four programmes was strengthening social support, for instance by encouraging family members to increase social networks. Behavioural activation was used in three programmes. Less frequent components were relaxation exercises and exposure techniques as a way to reduce anxiety.

### Recruitment

Table 4 describes the recruitment approaches. Most studies used multiple recruitment strategies. All but one study recruited participants via mental health care services. Specifically, three studies used databases from Health Maintenance Organization (HMO)^1^ databases, two studies had direct referrals from practitioners, four studies used letters to physicians and/or flyers in health clinics, and two studies did not specify how exactly participants were recruited via mental health clinics. In addition, seven studies used recruitment strategies via media outlets (e.g. newspapers, radio, television, and internet postings). The success of recruitment varied widely. There was a mean of 40.9% (range 2.8–78.4%) of families/offspring that participated in the trial after contact/eligibility assessment and 22.7% (range 1.7–78.3%) that actively refused to participate. Moreover, it took studies a mean of 2.1 years to recruit a mean of 133 families (63 families per year).^2^ For instance, despite being a multicentre study in four US cities, it took Garber et al. (2009) almost 3 years to recruit 316 offspring.

**Table 4:** Information on recruitment strategies and difficulties

Study main reference	Recruitment (% of families recruited from that source, if available)	How many were initially approached?	% of families contacted who also participated in trial	% of families who actively declined to participate	Time recruitment period	Remarks on recruitment difficulties
Beardslee (1997)	HMO (53.6%), mental health practitioners (13.05%), support groups (9.57%), advertisements (9.57%), other sources (18.26%)	Not reported	Not reported	Not reported	2.5 years (93 families)	Not reported
Clarke (2001)	HMO computerized pharmacy database for adults who had received at least 2 dispensations of an antidepressant within previous 12 months, the mental health appointment database was also searched for adults with at least two mental health visits in the past 12 months	3374 offspring & 2995 parents were sent letters	2.8% (offspring)	78.3% (families)	2 years (94 offspring)	The authors acknowledge that only a small number of identified subjects were enrolled out the pool of potential subjects. According to them, it raises concerns about patients’ interest in preventive services
Compas (2009)	Mental health clinics/practices (31%), family and general medical (9%) practices, media and public setting (53%), other (7%)	967 families contacted the research team	18.6% (families)	22.9% (families)	Not reported	Not reported
Garber (2009)	HMO computerized database; a university medical centre e‐mail listserv; letters to physicians in the community; letters to parents of students in local schools; and newspaper, radio, and television advertisements	2999 offspring screened for eligibility	10.5% (offspring)	11.3% (families)	3 years (316 offspring)	Not reported
Ginsburg (2009)	Advertisements in local papers, mailings to local physicians and psychiatrist, community flyers	51 families screened for eligibility	78.4% (families)	Not reported	Not reported	Not reported
Ginsburg (2015)	Advertisements in local papers, mailings to local physicians and psychiatrists, community flyers, radio advertisements	174 families completed baseline assessment	78.2% (families)	1.7% (families)	Not reported	Not reported
Goldstein (2018)	Outpatient psychiatric services, ongoing research studies, adult BP support groups, advertisements	68 families were contacted	62% (families)	22% (families)	Not reported	Offspring were more likely to decline compared to parents. In a pilot study, the authors reported a higher refusal rate (67%), because many offspring said they would not participate because there is nothing wrong with them. In the present trial, they highlighted that the intervention targets universal themes, which, according to the authors, led to a lower refusal rate
Mason (2012)	Flyers in health care clinics and therapeutic centres, internet postings, magazine advertisements, targeted letters, parenting seminars, and school contacts and presentations	51 families contacted the research team	58.8% (families)	Not reported	1 year (30 families)	Recruitment started with distribution of flyers in health care clinics and therapeutic centres. But due to slow rate of recruitment, strategies were expanded (see column recruitment)
Rasing (2017)	Schools	862 offspring assessed for eligibility	16.5% (offspring)	13.7% (offspring)	Not reported	Not reported
Solantaus (2010)	Health care units (Clinicians in the participating mental health units provided both verbal and written information of the study to the patients)	Not reported	40–45% (families, based on estimation from clinicians)	9.2% (families)	2 years (119 families)	Major reason for refusal were due to patients (35%; e.g. felt better, were not interested) and other family members not being willing to participate (40%)

The three studies that predominately relied on flyers and media outreach, where participants had to actively contact them, report high rates of families actually participating in the trial (>60%). Three studies that used HMO databases show relatively low participation rates. For instance, Clarke et al. (2001) used solely HMO databases based on antidepressants and mental health clinic visits and reported that only 2.8% of those approached participated in the trial with an active refusal rate of 78.3%. One study that was relatively successful with recruiting participants was the study of Solantaus, Paavonen, Toikka, and Punamäki (2010). The researchers recruited their participants via clinicians. Using this strategy, 40–45% of participants approached ended up participating in the trial.

Four studies gave some indication on whether they had difficulties with recruiting participants (see Table 4). For instance, Solantaus et al. (2010) stated that a major reason for refusal were due to patients (35%; e.g. felt better, were not interested) and other family members not being willing to participate (40%). Additionally, Goldstein et al. (2018) reported on a pilot trial in which the refusal rate was quite high (67%; Goldstein et al., 2014). In this trial, especially offspring declined participation, because they felt that ‘nothing was wrong’ with them. In their follow‐up trial (Goldstein et al., 2018), they highlighted to participants that the treatment targets more universal themes, which led, according to the authors, to a lower refusal rate (22%).

### Meta‐analysis results

**Figure 2:**
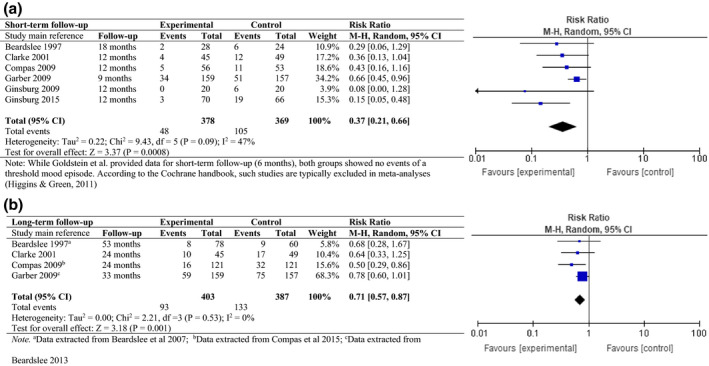
(a) Effect of prevention programme versus any control condition on the incidence of depression/anxiety disorder (short‐term follow‐up). (b) Effect of prevention programme versus any control condition on the incidence of depression/anxiety disorder (long‐term follow‐up).

**Figure 3:**
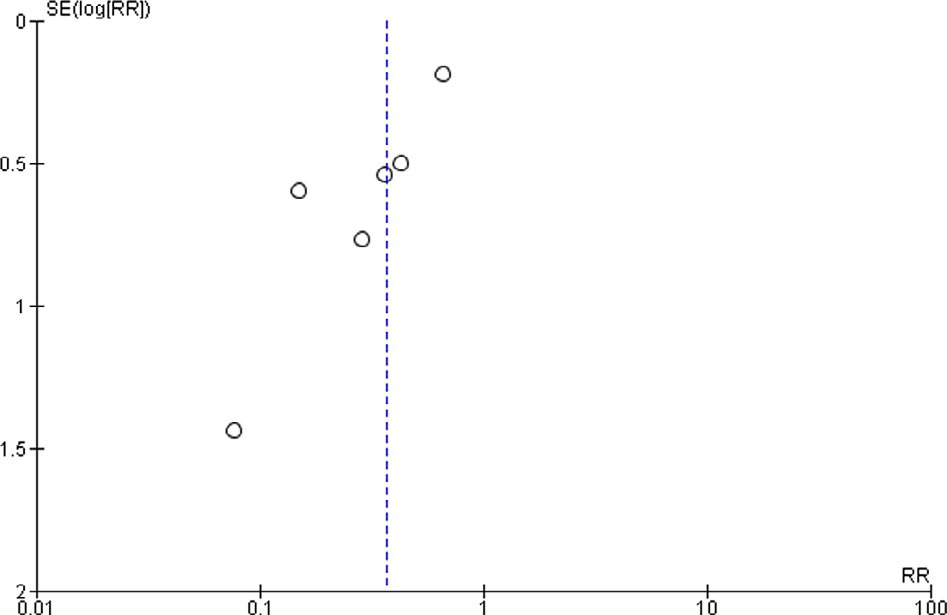
Funnel plot of incidence of depression/anxiety at 12‐month follow‐up.

Six of the ten studies reported data on depression/anxiety incidence. The meta‐analysis showed that the risk of developing a depressive/anxiety disorder was significantly reduced in children in the experimental condition as compared to those in the control condition at short‐term (RR = 0.37, 95% CI [0.21; 0.66], Figure 2a) and long‐term follow‐up (RR = 0.71, 95% CI [0.57; 0.87], Figure 2b). In other words, prevention programmes reduced the risk of a depressive/anxiety disorder in offspring by 63% after one year and by 29% after two years. This corresponds to a Number Needed to Treat (NNT) of 6.3 and 16.3, respectively. The *I* ^2^‐statistic suggests some heterogeneity for the 12‐month outcome (*I* ^2^ = 47%, *p* = .09). A funnel plot, as presented in Figure 3, shows slight asymmetry, indicating potential publication bias.

**Figure 4:**
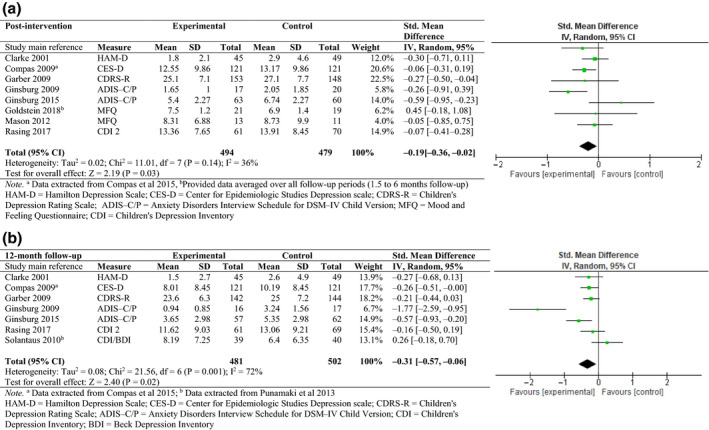
(a) Effect of prevention programme versus any control condition on depressive/anxiety symptoms (post‐intervention). (b) Effect of prevention programme versus any control condition on depressive/anxiety symptoms (12‐month follow‐up).

**Figure 5:**
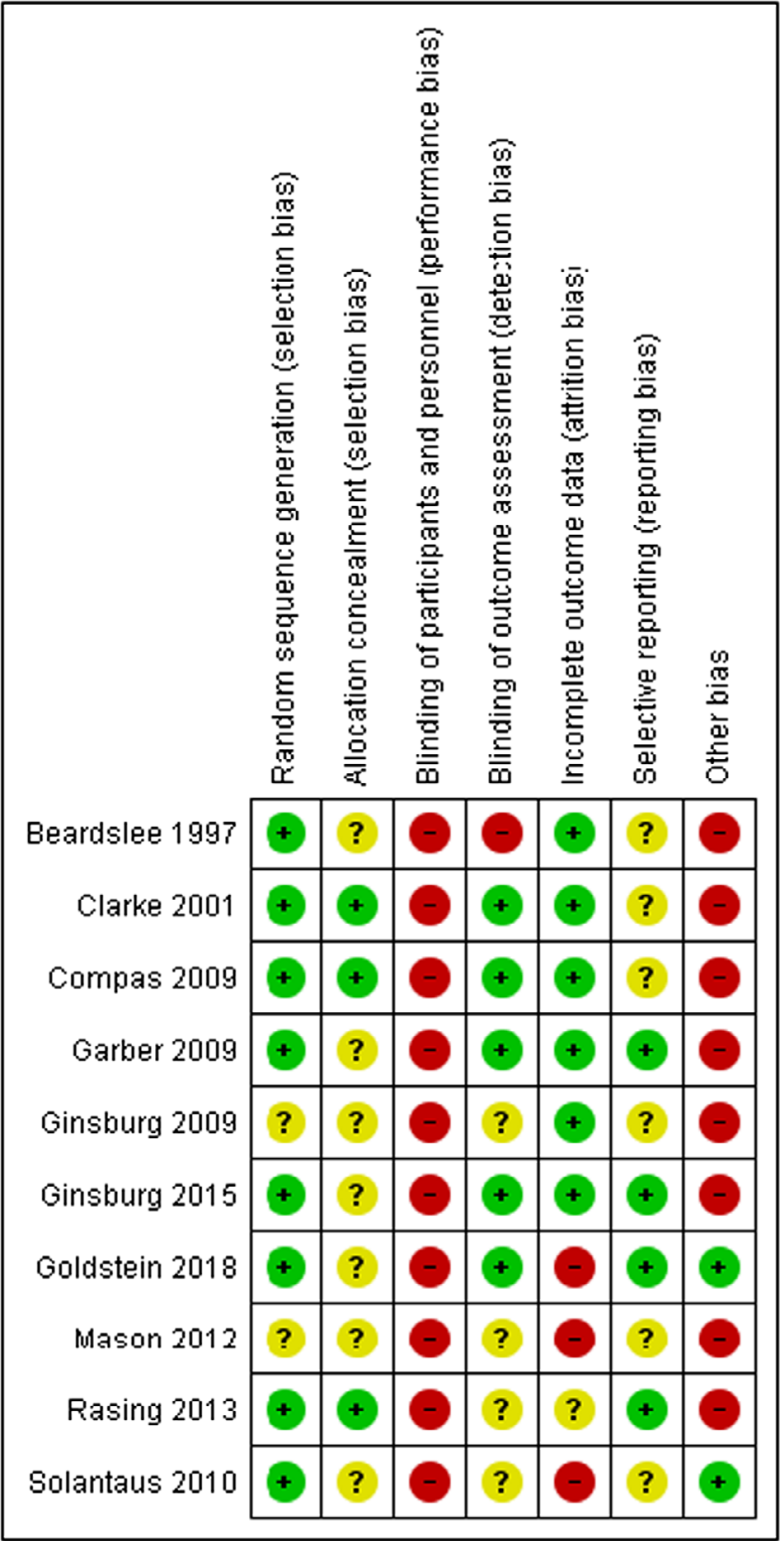
Risk of bias assessment across included studies.

Nine of ten studies reported data on mood/anxiety symptoms in offspring at post‐intervention and/or at 12‐months follow‐up. Results showed a significant difference between the experimental and control condition at post‐treatment (i.e. immediately after the intervention; SMD = −0.19, 95% CI [−0.36; −0.02], Figure 4a) and 12‐month follow‐up (SMD = −0.31, 95% CI [−0.57; −0.06], Figure 4b). Figure 5 shows the results of the risk of bias assessments, indicating that the quality of the studies varied greatly (see Appendix S3 for more information).

## Discussion

The present review provides a fine‐grained overview of the characteristics and techniques of prevention programmes for children of parents with mood/anxiety disorders. A total of 22 articles reporting on ten studies containing eight unique prevention programmes involving 1,325 subjects were identified, all delivered in face‐to face format directed to offspring or the family as a whole. Although these programmes varied in the number and types of techniques used, all contained a psychoeducational element. Studies differed in their way of recruiting participants. Results suggested that recruitment via clinicians was more successful than recruitment via Health Maintenance Organization databases. Positive, but small effects were found in favour of prevention programmes on the risk of developing a depressive/anxiety disorder (short‐term follow‐up RR = 0.37; long‐term follow‐up RR = 0.71) and mood/anxiety symptom levels (post‐intervention *g*′ = −0.19; 12‐month follow‐up *g*′ = −0.31).

### Characteristics of prevention programmes

The prevention programmes could roughly be divided into offspring‐focused and family‐focused interventions, the latter actively engaging both parents and children. All but one programme (Rasing et al., 2018) adopted a disorder‐specific approach. Substantial comorbidity rates between depressive and anxiety disorders led Rasing et al. (2018) to adopt a transdiagnostic approach. As no effects were found for this programme, the authors speculate that a potential mismatch between the exercises offered and offspring needs (e.g. exercises to reduce anxiety when no anxiety symptoms are experienced) may be reasons for a lack of intervention effect. They therefore argue that prevention programmes should have a clear focus on either depression or anxiety.

All prevention programmes were delivered in a face‐to‐face format. Given that online interventions show high accessibility and cost‐effectiveness (Donker et al., 2015) and that offspring seem to favour online interventions (Grové, Reupert, & Maybery, 2016), it remains to be determined whether delivering programmes in an online format are an alternative. Recent meta‐analyses emphasize that online interventions are effective in reducing internalizing problems in children/adolescents (Ebert et al., 2015; Pennant et al., 2015) and can be as effective as face‐to‐face treatments (Andersson & Titov, 2014; Vigerland et al., 2016). Thus, it may be worthwhile to put effort in further exploring online opportunities.

We used the TIDieR checklist (Hoffmann et al., 2014) to systematically map characteristics of the included prevention programmes. However, it became evident that published papers do not provide enough information to complete the TIDieR. For example, details on ‘intervention materials’ were commonly missing which is consistent with observations in other fields (Albarqouni, Glasziou, & Hoffmann, 2018; Hoffmann, Erueti, & Glasziou, 2013). Future studies should provide more detailed descriptions of interventions.

### Techniques used in prevention programmes

Echoing findings of previous studies focusing on prevention programmes for children of parents with mental disorders in general (Marston et al., 2016; Reupert & Maybery, 2010), all prevention programmes provided psychoeducation and on how parental illness may affect other family members. Studies have indicated that a lack of knowledge on parental mental illness could be a source of frustration and fears (Meadus & Johnson, 2000; Trondsen, 2012). Children note that improved understanding of their parents condition and its impact on parental behaviour could contribute to diminish such feelings (Beardslee et al., 1997). For example, a boy participating in Beardslee’s intervention noted that he ‘used to feel it's our fault for getting her angry (…), but now (we) know she has a problem. It's helped a lot to know this’. (Beardslee et al., 1997, p. 202) illustrating the importance of openness regarding parental psychopathology. The importance of linking psychoeducation on depression/anxiety to family experiences is supported by a recent review (Riebschleger, Grové, Cavanaugh, & Costello, 2017). In most programmes, psychoeducation mainly focused on parental disorders. Given the high comorbidity between depressive and anxiety disorder and non‐specific familial aggregation of psychiatric disorders (Dean et al., 2010; McLaughlin et al., 2012; Starr, Conway, Hammen, & Brennan, 2014), limiting psychoeducational efforts to the specific parental disorder may be a missed opportunity.

Cognitive restructuring techniques can be considered a central component of the offspring‐focused interventions. Such techniques are among the basic tenets of cognitive‐behavioural therapy and commonly used in depression/anxiety treatment. McLaughlin (2011) suggests that this technique may be particularly useful for persons already suffering from increased symptom levels. In persons with few symptoms, these distortions may not be present and as such these techniques may be difficult to internalize. Studies indicate that prevention programmes may be more beneficial for offspring with higher baseline severity levels (Bettis, Forehand, Sterba, Preacher, & Compas, 2018; Ginsburg, Drake, Tein, Teetsel, & Riddle, 2015); however, as programmes have been evaluated as a whole, it remained unclear whether this cognitive restructuring technique was a contributing factor herein.

In contrast to offspring‐focused prevention programmes, family‐focused programmes share a focus on family environment factors thought to be linked to the intergenerational transmission of mood/anxiety disorders (i.e. parenting skills and family communication; Beardslee, Gladstone, & O’Connor, 2011; Creswell & Waite, 2015). For example, parenting behaviours typical for anxious parents (e.g. parental overprotection) were targeted in the Coping and Promoting Strengths programme (Ginsburg, 2009; Ginsburg et al., 2015). Additionally, in the Family Group Cognitive‐Behavioral Intervention (Compas et al., 2009), parents learned to praise children and encourage them to use coping skills. Family communication was addressed by improving general communication between family members (e.g. teaching active listening) as well as communication related to parental illness. The latter is a central aim of Hope, Meaning, and Continuity (Beardslee et al., 1997) where the development of a shared understanding of parental illness involving individual experiences of each of the family members is considered one of the central ‘healing’ principles. Indeed, studies suggest that targeting family‐related factors to prevention programmes may facilitate stronger outcomes in children (e.g. Collins & Dozois, 2008; Thanhäuser et al., 2017; Van Santvoort, Hosman, Van Doesum, & Janssens, 2013).

In addition to aforementioned central components of offspring‐ and family‐focused interventions, the programmes vary in the number and type of additional components they contain. Four programmes teach offspring problem‐solving skills, which can help offspring to better cope with everyday problems (whether or not related to parental illness) that may hamper optimal emotional functioning. Prevention programmes focusing on prevention of anxiety disorders additionally include exposure techniques, which is a well‐established treatment for anxiety (Kaczkurkin & Foa, 2015). Rasing, Creemers, Janssens, and Scholte (2017) underline the importance of using exposure techniques also in the context of prevention given the relatively high symptom levels already experienced by high‐risk adolescents likely requiring strong enough techniques to bring about change. Behavioural activation techniques were implemented in three programmes. Although behavioural activation is widely used as treatment strategy in adults (Boswell, Iles, Gallagher, & Farchione, 2017; Cuijpers, van Straten, & Warmerdam, 2007), there is yet limited evidence to support its use in children/adolescents, although initial findings are promising (Martin & Oliver, 2019). Lastly, social support positively influences mental health and well‐being (Newman, Newman, Griffen, O’Connor, & Spas, 2007; Umberson & Karas Montez, 2010) and has been identified by offspring as resource that help them to cope with experiences related to parental illness (Beardslee & Podorefsky, 1989; Drost, van der Krieke, Sytema, & Schippers, 2016). However, strengthening social support was addressed in only three programmes. Relaxation techniques were not frequently implemented, although research shows that it may help in diminishing mood symptoms (Jorm, Morgan, & Hetrick, 2008).

All prevention programmes combined psychoeducational elements with skills training and/or cognitive‐behavioural therapy elements. The Coping and Promoting Strengths programme combined all ten techniques we identified in our content analysis. This programme also showed the largest effect sizes. It is however unclear whether the inclusion of multiple intervention techniques was responsible for these beneficial effects. Thus, little is known about the specific effects of the different components used in prevention programmes. This is an important area for future research.

### Recruitment difficulties

We additionally analysed recruitment strategies and difficulties. However, we note that not all studies provided sufficient information on this; thus, conclusions should be treated with caution. Most studies used advertisements and media outlets for recruiting their participants. Here, a large number of people approaching the research team actually ended up participating in the trial but it remains unclear how many people were reached and decided to contact the research team. Naturally, those that contacted the research team are likely to be interested in participating. Studies using HMO databases, for example the study of Clarke et al. (2001), reported low participation and high refusal rates. In contrast, Solantaus et al. (2010) recruited their participants via clinicians and reported a low refusal rate. It could be that participants trust their clinicians more when they inform them about potential studies compared to when they just receive ‘impersonal’ letters. Based on our results, we recommend two things: first, studies should employ multiple recruitment strategies to increase chances of recruitment success. Second, based on success recruitment rates from different studies, we suggest that studies should try to include participants in a more personal way, for instance via clinicians with whom the participants already have a relationship.

In general, even Solantaus et al. (2010), who were relatively successful compared to other studies, reported on recruitment difficulties supporting the view that this population may be difficult to engage in research. It appears more challenging to enrol participants in prevention than in treatment trials, probably because treatment trials offer benefits to an active medical problem while prevention trials offer the possibility of prevention of potential future, but maybe not yet existing problems (Cooper et al., 2015; Spilker & Cramer, 1992). In parents with depression/anxiety in particular, parental overburden, shame and stigma, and perceived lack of necessity for intervention were important reasons to refuse participation in an offspring prevention trial which was ended preliminary due to a lack of participants (Festen et al., 2014; Nauta et al., 2012). To what extent the participants in prevention programmes are representative of the entire population of children of parents with mood/anxiety disorders remains to be determined.

An accurate description of the recruitment process is often lacking in RCTs (Gross, Mallory, Heiat, & Krumholz, 2002), but of high importance for several reasons: it is helpful to optimize recruitment strategies in future studies, informs us about the generalizability of study results, and aids in taking informed decisions on whether or not to start a trial or to implement a prevention programme. The latter is, for example, less attractive when, in spite of shown benefits, the target group is hard to reach. As this issue is relevant to the broader field of intervention research, we recommend to consider to include this topic more explicitly in the TIDieR checklist. Quantitative (e.g. number of participants contacted) and qualitative information (e.g. reasons for refusal) is likely to be relevant here.

### Meta‐analysis effect of prevention programmes

Our meta‐analytic results show that prevention programmes for children of parents with mood/anxiety disorders reduce children’s risk of developing depressive/anxiety disorders and decrease symptom levels at short‐term and long‐term follow‐up. This is in accordance with studies on the efficacy of prevention programmes focusing on parental mental illness in general (Siegenthaler et al., 2012; Thanhäuser et al., 2017) and parental depression in particular (Loechner et al., 2018). These meta‐analyses found, like us, small, but significant beneficial effects for child outcomes. Note that while there was overlap between the studies included in the previous meta‐analyses, there were also four unique trials that were included in our meta‐analysis that were not included in the meta‐analysis by Loechner et al., (2018). Moreover, while Thanhäuser et al. (2017) included a large amount of studies, it is unclear whether effect were specific to depression and anxiety. Additionally, the meta‐analysis only focused on psychopathology symptoms in children and not on incidence rates.

Our results indicate significant long‐term effects on the incidence of depression/anxiety, but the magnitude appears to diminish over time. In contrast to our results, Loechner et al. (2018) found only post‐intervention but not short‐term and long‐term effects on the severity of depressive symptoms. In contrast to Loechner et al. (2018), we additionally focused on parental bipolar disorder and anxiety disorder and included additionally four other trials which could be an explanation for the difference in findings. Indeed, two studies focusing on anxiety prevention had very positive results (Ginsburg, 2009; Ginsburg et al., 2015). It is possible that interventions for offspring of parents with anxiety are more effective. However, research indicates that cognitive‐behavioural treatments for anxiety and depression in children/adolescents show similar effect sizes (Crowe & McKay, 2017). Alternatively, these two studies included most techniques and might thus have been more powerful than the other programmes (Ginsburg, 2009; Ginsburg et al., 2015). As mentioned earlier, we could not verify this, because programmes have been evaluated as a whole. Due to the relatively small sample size of trials, we were unable to assess potential moderators and mediators for treatment efficacy. In fact, only half of the trials assessed moderators and/or mediators and those factors that were investigated differed substantially across studies. For instance, some studies evaluated whether parental and offspring symptom severity at baseline were moderators. However, results were conflicting. Whereas Weersing et al. (2016) found that offspring whose parents were depressed at baseline benefitted *less* from the intervention, Compas et al. (2011) found that parental depression at baseline did not moderate intervention effects. Moreover, while Ginsburg et al. (2015) found that offspring with higher symptom severity at baseline showed greater improvements in symptoms in response to the interventions, Weersing et al., (2016) showed that intervention effects were diminished for offspring with higher symptom severity. Moreover, mediators differed heavily across interventions. Some of the variables that have been shown to explain intervention effects were *individual* factors, such as coping (Compas et al., 2010) and positive attribution (Punamaki, Paavonen, Toikka, & Solantaus, 2013), and *parental* factors, such as parental monitoring (Ginsburg et al., 2015) and positive parenting (Compas et al., 2010). Due to the non‐systematic assessment of moderators and mediators in the included trials, we were unable to run sensitivity analyses regarding these effects. A systematic assessment of intervention moderators and mediators is clearly needed to better establish why prevention programmes work and for whom they work best. Individual patient‐data meta‐analyses would be equipped to better understand influences of such moderators and mediators.

### Limitations

There are several limitations that need to be acknowledged when interpreting the results of this study. First, the description of the Family Group Cognitive‐Behavioral Intervention (Compas et al., 2009) was entirely based on information provided in published research papers as the programme manual was not available. Second, recruitment approaches and difficulties were insufficiently described hampering us to draw firm conclusions on this issue. Third, due to the limited number of prevention programmes included in our review, we were not able to assess which specific intervention techniques were related to programme efficacy. Fourth, there is likely to be a selection bias in the trials. It could for instance be that especially parents that are aware of the risk for their children participate. On the other hand, it could also be that parents and offspring with high psychopathology are more reluctant to participate, because they do not have the energy to participate (Festen et al., 2014; Wals et al., 2001). Additionally, those parents who feel better after finishing their treatments might not be interested (Solantaus et al., 2010), because they do not want to be confronted with their disorder again. Fifth, the follow‐up period was limited and long‐term benefits of prevention programmes remain to be determined. Finally, the present review was not pre‐registered. However, we tried to conduct the present review as objectively as possible with two independent assessors.

### Conclusion

The prevention programmes for children of parents with mood/anxiety disorders included in the present review combined psychoeducational elements with skill training and/or cognitive‐behavioural therapy elements. Our meta‐analysis suggests that prevention programmes are effective in reducing the risk for developing a mood/anxiety disorder in offspring. Despite these promising results, we know little about which specific intervention components contribute to these beneficial outcomes as little attention has yet been given to the individual components making up these prevention programmes. Thus, future studies of sufficient power to detect effective components are required to achieve a better understanding of the active components of these prevention programmes. Such studies can help to improve the efficacy of prevention programmes and to successfully integrate them into clinical practice (IOM, 2015). Future studies should also address mediators and moderators for their treatments, so that we can learn how and for whom interventions work. Moreover, to increase recruitment success, studies should use multiple recruitment strategies. Based on our review, it seemed that recruitment via clinicians has the best chances of success. Last but not least, efforts should be made to improve the completeness of intervention descriptions in future efficacy trials. Specifically, researchers should make a greater effort in reporting on the content of their interventions (e.g. using the TIDieR checklist), make their manuals available, and also be more complete about their recruitment strategies and difficulties. This information contributes to a solid basis for future investigations and could improve the uptake of research findings into clinical practice.

## Conflicts of interest

All authors declare that they have no conflicts of interest.

## Author contributions

Petra Havinga (Conceptualization; Formal analysis; Investigation; Methodology; Writing – original draft; Writing – review & editing) Dominique Maciejewski (Conceptualization; Formal analysis; Methodology; Supervision; Writing – original draft; Writing – review & editing) Catharina Hartman (Writing – review & editing) Manon Hillegers (Conceptualization; Funding acquisition; Writing – review & editing) R.A Schoevers (Writing – review & editing) Brenda Penninx (Conceptualization; Funding acquisition; Writing – review & editing).

## Supporting information


**Appendix S1**. Keywords used for literature searches in Pubmed, Psycinfo, and Central.
**Appendix S2**. TIDieR checklist for control conditions.
**Appendix S3**. Risk of bias assessment across the preventive intervention studies.

## Data Availability

Research data are not shared, because this is a systematic review and we did not have access to the data of the individual studies.

## Footnotes

^1^ HMOs are medical insurance groups, for which individuals pay a monthly fee for comprehensive health care services. The HMO databases include information about these individuals (e.g. information about anti‐depressant usage).

^2^ Note that not all studies indicated the time window of recruitment.
