# Supplementary material for: Prevention programmes for children of parents with a mood/anxiety disorder: Systematic review of existing programmes and meta‐analysis of their efficacy
Source: Br J Clin Psychol. 2021 Jan 6;60(2):212–51. doi: 10.1111/bjc.12277 (PMC8248072; doi:10.1111/bjc.12277)
Supplement: Supplementary file 1 — Appendix S1. Keywords used for literature searches in Pubmed, Psycinfo, and Central. Appendix S2. TIDieR checklist for control conditions. Appendix S3. Risk of bias assessment across the preventive intervention studies. [file BJC-60-212-s001.docx]

**Appendix Paper: Prevention programs for children of parents with a mood/anxiety disorder: Systematic review of existing programs, and meta-analysis of their efficacy**

**Appendix S1:** Keywords used for literature searches in Pubmed, Psycinfo, and Central

|  | Pubmed | Psycinfo | Central |
| --- | --- | --- | --- |
| Keywords for children of disordered parents | (("Child of Impaired Parents"[Mesh] OR COPMI [tiab] OR impaired parent* [tiab] OR impaired mother* [tiab] OR mentally ill parent* [tiab] OR mentally ill mother* [tiab] OR parental illness* [tiab] OR maternal illness [tiab] OR paternal illness [tiab] OR bipolar offspring [tiab] OR depressed offspring [tiab] OR parental psychopatholog* [tiab] OR maternal psychopatholog* [tiab] OR paternal psychopatholog* [tiab] OR maternal depress* [tiab] OR paternal depress* [tiab] OR parental depress* [tiab] OR maternal anxiet* [tiab] OR paternal anxiet* [tiab] OR parental anxiet* [tiab] OR depressed parent* [tiab] OR depressed mother* [tiab] OR depressed father* [tiab] OR anxious parent* [tiab] OR anxious mother* [tiab] OR anxious father* [tiab] OR bipolar parent* [tiab] OR bipolar mother* [tiab]) OR (("Depression"[Mesh] OR "Mood Disorders"[Mesh] OR depression* [tiab] OR depressed* [tiab] OR depressive* [tiab] OR dysthymi* [tiab] OR melanchol* [tiab] OR affective disorder* [tiab] OR "Anxiety Disorders"[Mesh] OR “Anxiety” [Mesh] OR anxiet* [tiab] OR anxious* [tiab] OR agoraphobi* [tiab] OR panic* [tiab] OR phobi* [tiab] OR "Bipolar and Related Disorders"[Mesh] OR bipolar [tiab] OR manic* [tiab] OR mania* [tiab] OR hypomania* [tiab] OR hypomanic [tiab]) AND (family histor* [tiab] OR familial histor* [tiab] OR family risk* [tiab] OR familial risk* [tiab] OR familial transmission* [tiab] OR family transmission* [tiab] OR at-risk child* [tiab] OR at-risk offspring [tiab] OR at-risk adolescent* [tiab] OR at-risk youth* [tiab] OR child at-risk [tiab] OR adolescents at-risk [tiab] OR children at risk [tiab] OR offspring at-risk [tiab] OR youth at-risk [tiab] OR high-risk child* [tiab] OR high-risk adolescent* [tiab] OR high-risk offspring [tiab] OR high-risk youth* [tiab]))) | DE "Offspring" OR DE "Transgenerational Patterns" OR TI(COPMI OR impaired parent* OR impaired mother* OR impaired father* OR mentally ill parent* OR mentally ill mother* OR mentally ill father* OR parental illness OR maternal illness OR paternal illness OR bipolar offspring OR depressed offspring OR anxious offspring OR parental psychopatholog* OR maternal psychopatholog* OR paternal psychopatholog* OR maternal depress* OR paternal depress* OR parental depress* OR maternal anxiet* OR paternal anxiet* OR parental anxiet* OR depressed parent* OR depressed mother* OR depressed father* OR anxious parent* OR anxious mother* OR anxious father* OR bipolar parent* OR bipolar mother* OR bipolar father* OR maternal bipolar OR paternal bipolar OR parental bipolar) OR AB(COPMI OR impaired parent* OR impaired mother* OR impaired father* OR mentally ill parent* OR mentally ill mother* OR mentally ill father* OR parental illness OR maternal illness OR paternal illness OR bipolar offspring OR depressed offspring OR anxious offspring OR parental psychopatholog* OR maternal psychopatholog* OR paternal psychopatholog* OR maternal depress* OR paternal depress* OR parental depress* OR maternal anxiet* OR paternal anxiet* OR parental anxiet* OR depressed parent* OR depressed mother* OR depressed father* OR anxious parent* OR anxious mother* OR anxious father* OR bipolar parent* OR bipolar mother* OR bipolar father* OR maternal bipolar OR paternal bipolar OR parental bipolar)  OR  ((DE "Anaclitic Depression" OR DE "Dysthymic Disorder" OR DE "Endogenous Depression" OR DE "Late Life Depression" OR DE "Postpartum Depression" OR DE "Reactive Depression" OR DE "Recurrent Depression" OR DE "Treatment Resistant Depression" OR DE "Depression (Emotion)" OR DE "Major Depression" OR DE "Mania" OR DE "Affective Disorders" OR DE "Hypomania" OR DE "Bipolar Disorder" OR DE "Anxiety" OR DE "Anxiety Disorders" OR DE "Generalized Anxiety Disorder" OR DE "Panic Disorder" OR DE "Phobias" OR TI(depression* OR depressed* OR depressive* OR dysthymi* OR melanchol* OR affective disorder* OR anxiet* OR anxious* OR agoraphobi* OR panic* OR phobi* OR bipolar OR manic* OR mania* OR hypomania* OR hypomanic) OR AB(depression* OR depressed* OR depressive* OR dysthymi* OR melanchol* OR affective disorder* OR anxiet* OR anxious* OR agoraphobi* OR panic* OR phobi* OR bipolar OR manic* OR mania* OR hypomania* OR hypomanic)) AND (DE "At Risk Populations" OR TI (family histor* OR familial histor* OR family risk* OR familial risk* OR familial transmission* OR family transmission* OR at-risk child* OR at-risk offspring OR at-risk adolescent* OR at-risk youth* OR high-risk child* OR high-risk adolescent* OR high-risk offspring OR high-risk youth*) OR AB(family histor* OR familial histor* OR family risk* OR familial risk* OR familial transmission* OR family transmission* OR at-risk child* OR at-risk offspring OR at-risk adolescent* OR at-risk youth* OR high-risk child* OR high-risk adolescent* OR high-risk offspring OR high-risk youth*)) | (COPMI OR impaired parent* OR impaired mother* OR mentally ill parent* OR mentally ill mother* OR parental illness* OR maternal illness OR paternal illness OR bipolar offspring OR depressed offspring OR parental psychopatholog* OR maternal psychopatholog* OR paternal psychopatholog* OR maternal depress* OR paternal depress* OR parental depress* OR maternal anxiet* OR paternal anxiet* OR parental anxiet* OR depressed parent* OR depressed mother* OR depressed father* OR anxious parent* OR anxious mother* OR anxious father* OR bipolar parent* OR bipolar mother*)  OR  (depression* OR depressed* OR depressive* OR dysthymi* OR melanchol* OR affective disorder* OR anxiet* OR anxious* OR agoraphobi* OR panic* OR phobi* OR bipolar OR manic* OR mania* OR hypomania* OR hypomanic) AND (family histor* OR familial histor* OR family risk* OR familial risk* OR familial transmission* OR family transmission* OR at-risk child* OR at-risk offspring OR at-risk adolescent* OR at-risk youth* OR child at-risk OR adolescents at-risk OR children at risk OR offspring at-risk OR youth at-risk OR high-risk child* OR high-risk adolescent* OR high-risk offspring OR high-risk youth*) |
| Keywords for Prevention – intervention | (“Primary Prevention” [Mesh] OR “Preventive psychiatry” [Mesh] OR "prevention and control" [Subheading] OR prevent* [tiab] OR interven* [tiab] OR psychoeducati* [tiab] OR psycho-educati* [tiab] OR "Telemedicine"[Mesh] OR ehealth [tiab] OR e-health [tiab] OR mhealth [tiab] OR m-health [tiab] OR "Mobile Applications"[Mesh] OR mobile app* [tiab]) | DE "Preventive Medicine" OR DE "Primary Mental Health Prevention" OR DE "Group Intervention" OR DE "School Based Intervention" OR DE "Prevention" OR DE "Crisis Intervention" OR DE "Intervention" OR DE "Suicide Prevention" OR DE "Family Intervention" OR DE "Psychoeducation" OR DE "Early Intervention" OR DE "Online Therapy" OR DE "Mobile Devices" OR TI(prevent* OR interven* OR psychoeducati* OR psycho-educati* OR ehealth OR e-health OR mhealth OR m-health OR mobile app* OR mobile device*) OR AB(prevent* OR interven* OR psychoeducati* OR psycho-educati* OR ehealth OR e-health OR mhealth OR m-health OR mobile app* OR mobile device*) | prevent* OR interven* OR psychoeducati* OR psycho-educati* OR ehealth OR e-health OR mhealth OR m-health OR mobile app* OR mobile device* |
| Keywords for RCT/ Clinical trial | (random*[tiab] AND (controlled[tiab] OR control[tiab] OR placebo[tiab] OR versus[tiab] OR vs[tiab] OR group[tiab] OR groups[tiab] OR comparison[tiab] OR compared[tiab] OR arm[tiab] OR arms[tiab] OR crossover[tiab] OR cross-over[tiab]) AND (trial[tiab] OR study[tiab])) OR ((single[tiab] OR double[tiab] OR triple[tiab]) AND (masked[tiab] OR blind*[tiab])) OR (randomized controlled trial[pt] OR controlled clinical trial[pt] OR clinical trial [pt] OR clinical trial* [tiab]) | DE “Clinical Trials” OR DE “Treatment Effectiveness Evaluation” OR TI(clinical trial* OR controlled trial* OR randomized trial* OR randomised trial* OR randomized stud* OR randomised stud* OR RCT) OR AB(clinical trial* OR controlled trial* OR randomized trial* OR randomised trial* OR randomized stud* OR randomised stud* OR RCT) | randomized or randomised or controlled |

**Appendix S2:** TIDieR checklist for control conditions

| Name of control condition | Why | What (Materials) | What (Procedures) | Who provided | How |
| --- | --- | --- | --- | --- | --- |
| Lecture intervention  *Beardslee et al., 1997* | The central goals of this lecture intervention are to increase parental knowledge about the cause and symptoms of depression, to facilitate family discussion of parental affective illness and its impact on the family and to help parents identify and foster healthy coping strategies in their children. The lecture intervention is based on the same constructs as the clinican-facilitated intervention, but there is no attempt to link psychoeducational material to the family's illness experience. | Lecture, group discussion,  psychoeducational written materials for families, videotapes | Lecture is used to deliver psychoeducational material.  Materials describe depression, the state-of-the-art standard treatments, some of first authors own work on resilience, and other matters.  Time is available for questions and group discussions.  Videotapes are used for parents unable to attend the lectures with possibilities for additional consultation. | The first author delivered each lecture. | Face-to-face with group of parents (average three families). |
| Usual care  *Clarke et al., 2001* | All enrolled adolescents, regardless of randomization condition, were permitted to initiate or continue any non-study related mental health or other health services. | N/A | N/A | N/A | N/A |
| Written information self-study condition  *Compas et al., 2009* | To educate families about the nature of depression, the effects of parental depression on families, and the signs of depression in children. | Written psychoeducational material developed for parents and children separately (materials for children were based on age 9-11 or 12-15), schedule | To educate families, written materials are emailed to families (self-study).  Families are provided with a schedule for reading the materials. | Not described | Materials are e-mailed to families. Families had to study the materials themselves. |
| Usual care  *Garber et al., 2009* | All enrolled adolescents, regardless of randomization condition, were permitted to initiate or continue non-study related mental health or other health care services. | N/A | N/A | N/A | N/A |
| Waitlist control condition  *Ginsburg et al., 2009* | The waitlist constitutes the control condition. Participants could participate in intervention after the end of the study. | N/A | N/A | N/A | N/A |
| Information monitoring condition  *Ginsberg et al., 2015* | To educate adolescents about anxiety disorders and associated treatments. No detailed information about anxiety reduction strategies included in the intervention condition (CAPS) was given. | Educational pamphlet (36 pages) | The pamphlet is used to provide adolescents with information. | Not described | Families are given an educational pamphlet, which adolescents had to study individually. |
| Data-informed referral  *Goldstein et al. 2018* | (1) Review of ﬁndings from the intake clinical assessment (i.e., diagnoses and symptoms); (2) Review of ﬁndings from the intake sleep assessment, including the computer-generated actigraphy report; (3) Provision of DIR-clinically-indicated referrals for treatment based on data gathered from intake assessment. | None | N/A | N/A | N/A |
| Waitlist control condition  *Mason et al., 2012* | The waitlist constitutes the control condition. Participants could participate in intervention after the end of the study. | N/A | N/A | N/A | N/A |
| Waitlist control condition  *Rasing et al., 2017* | The waitlist constitutes the control condition. Participants could participate in intervention after the end of the study. | N/A | N/A | N/A | N/A |
| Brief psychoeducational  discussion with parents (Let’s Talk about Children, LT)  *Solantaus et al., 2010* | The intervention aims to assess the child’s situation and to provide information on how parents can support their children. | Discussion, self-help guide called ‘How Can I Help My Children, A Guide Book for Parents with Mental Health Problems’, a standard information booklet about depression. | The discussion is conducted with the patient and possibly with his/her partner to assess the child’s situation and to provide information on how parents can support their children.  The self-help guide and standard information booklet is used to deliver the psychoeducational material. | Clinicians carried out the intervention with their own patients. | Face-to-face with the patient and possibly his/her partner. |

| *Table 2 (continued).* TIDieR checklist for control conditions | | | | | | | |
| --- | --- | --- | --- | --- | --- | --- | --- |
| Name of control condition | Where | When and How Much | Tailoring | Modifications | How well (planned) | How well (actual) |  |
| Lecture intervention  *Beardslee et al., 1997* | Not described | Two separate lectures (each 1 hour) | Not described | Not described | A manual-based lecture script was used.  A standardized scale based on content information from the lecture script is used to measure adherence to the lecture script. A rater, blind to the family’s response to the lecture, rated 5 videotapes from the first lecture and 5 videotapes from the second lecture, randomly selected. | Adherence to the lecture protocol was >95%. |  |
| Usual care  *Clarke et al., 2001* | N/A | N/A | N/A | N/A | N/A | N/A |  |
| Written information self-study condition  *Compas et al., 2009* | Families could choose where to read the material | Materials were sent in three sets over an 8-week interval. | Not described | Not described | Research assistants checked with the families to ensure that they received the materials. In addition, families were provided with a schedule for reading the materials. | Not described |  |
| Usual care  *Garber et al., 2009* | N/A | N/A | N/A | N/A | N/A | N/A |  |
| Waitlist control condition  *Ginsburg et al., 2009* | N/A | N/A | N/A | N/A | N/A | N/A |  |
| Information monitoring condition  *Ginsberg et al., 2015* | Families could choose where to read the pamphlet | Not described | Not described | Not described | Not described | Not described |  |
| Data-informed referral  *Goldstein et al. 2018* | Not described | 1 session (45 minutes) | Not described | Not described | Not described | Not described |  |
| Waitlist control condition  *Mason et al., 2012* | N/A | N/A | N/A | N/A | N/A | N/A |  |
| Waitlist control condition  *Rasing et al., 2017* | N/A | N/A | N/A | N/A | N/A | N/A |  |
| Brief psychoeducational  discussion with parents (Let’s Talk about Children, LT)  *Solantaus et al., 2010* | Not described | Discussion time minimum 15 min and maximum two 45-min sessions | Not described | Not described | Discussion is manualized. | Not described |  |

*N/A = item not applicable for the control condition being describe*

**Appendix S3:** Risk of bias assessment across the preventive intervention studies

|  | Random sequence generation | Allocation concealment (selection bias) | Blinding of participants and personnel (performance bias) | Blinding of outcome assessment (detection bias) | Incomplete outcome data (attrition bias) | Selective reporting (reporting bias) | Other bias |
| --- | --- | --- | --- | --- | --- | --- | --- |
| Beardslee et al. (1997) | Low risk  "Pilot families were randomized to treatment groups on a 2/3:1/3 basis (clinician facilitated: lecture). We used a balanced block randomization procedure for the other 84 families, with blocks of 4 stratified by family type (single parent or dual parent; Beardslee et al., 1999)." (Beardslee et al., 2007, p. 705) | Unclear risk  No information specified | High risk  Blinding not possible due to the nature of the intervention | High risk  "It was impossible for individual assessors to be blind to group status, although child and parent assessors were kept blind to assessment information from each other." (Beardslee et al. 2007, p.705) | Low risk  * intention to treat design/statistical method to account for missing data: LOW RISK ("We performed regression analyses with SAS Version 9.0 using generalized estimating equations, which provide unbiased estimates of treatment effects and tests of hypotheses when data are missing at random" (Beardslee et al., 2007, p.707)  * Difference in drop-out between groups: LOW RISK ("The 8 families who dropped out between assessment and intervention were not different from the 97 families who completed the intervention on measures of global functioning or socioeconomic status. There were no significant differences between groups for completion status on any variable." (Beardslee et al., 2007, p.708))  * Missingness related to outcome: LOW RISK ("The 8 families who dropped out between assessment and intervention were not different from the 97 families who completed the intervention on measures of global functioning or socioeconomic status. There were no significant differences between groups for completion status on any variable." (Beardslee et al., 2007, p.708)) | Unclear risk  Protocol not available | High risk  * Trial conducted by those who developed the intervention.  * Inclusion criteria were not completely strictly executed: "Children were excluded from the study when parents reported a history of major depression in the children by brief screening. However, data from the Schedule for Affective Disorders and Schizophrenia for School-Age Children revealed that 16 children had experienced an episode of major depression prior to baseline." (Beardslee et al., 2007, p.710) |
| Clarke et al. (2001) | Low risk  "Qualifying subsyndromal youth were randomized to conditions using a block procedure to ensure that group assignments were never significantly imbalanced". (Clarke et al., 2001, p.1129) | Low risk  "Group assignment was preprinted using a computer program and sealed in sequentially numbered envelopes which were opened in sequential order by the project coordinator". (Clarke et al., 2001, p.1129) | High risk  Blinding not possible due to the nature of the intervention | Low risk  “Assessors were unaware of the experimental condition of interviewed subjects” (Clarke et al., 2001, p.1128) | Low risk  * intention to treat design/statistical method to account for missing data: LOW RISK (used an intent-to-treat design)  * Difference in drop-out between groups: UNCLEAR RISK (no test reported whether missing data differed between conditions)  * Missingness related to outcome: LOW RISK ("We found few baseline or treatment interaction differences between participating subjects and those unavailable for follow-up at any follow-up point on any of the key demographic, major affective, or psychopathological measures. None of the few differences were consistent across time, suggesting that there was no systematic bias in drop-out. As a further check on bias as a result of attrition, primary outcome analyses were conducted that included only participants who completed all 4 assessments. The patterns of results did not change when the sample was limited in this way, lending further confidence that missing data did not bias results". (Clarke et al., 2001, p.1130)) | Unclear risk  Protocol not available | High risk  Trial conducted by those who developed the intervention. |
| Compas et al. (2009) | Low risk  "The order of randomization was determined by a random number generator, and the assignment order was kept in a series of sealed envelopes that were opened by research assistants who were blind to assignment until the envelope was opened for a family." (Compas et al. 2015, p.546) | Low risk  "The order of randomization was determined by a random number generator, and the assignment order was kept in a series of sealed envelopes that were opened by research assistants who were blind to assignment until the envelope was opened for a family." (Compas et al. 2015, p.546) | High risk  Blinding not possible due to the nature of the intervention | Low risk  "Doctoral students in clinical psychology and staff research assistants, who were blind to condition, conducted the structured diagnostic interviews after receiving extensive training." (Compas et al., 2015, p.544) | Low risk  * intention to treat design/statistical method to account for missing data: LOW RISK ("Using an intent-to-treat approach, we fit all models with SAS 9.3 Proc Mixed and restricted maximum likelihood estimation (i.e., method = REML), which allows missing outcomes under missing-at-random assumptions and can provide less biased estimates of the variance components when the number of groups is small. (Compas et al. 2015, p.546))"  * Difference in drop-out between groups: LOW RISK ("Third, because not all parents and children provided complete data at all five time points, a variable reflecting the amount of missing data was derived and participants assigned to the FGCB intervention and the WI comparison condition were compared on this variable. The amount of missing data (i.e., missing data at none, one, two, three, four or five of the follow-up assessments) did not differ between families assigned to the FGCB condition vs. WI condition." (Compas et al. 2015, p.547))  * Missingness related to outcome: UNCLEAR RISK (not reported) | Unclear risk  Protocol not available | High risk  Trial conducted by those who developed the intervention. |
| Garber et al (2009) | Low risk  “Adolescents were randomized using the Begg and Iglewicz modification of the Efron biased coin toss to ensure that the 2 cells were balanced on age, sex, race/ethnicity, and inclusion criteria (i.e., history of depressive episode, high CES-D score)." (Garber et al., 2009; p.2217) | Unclear risk  No information specified | High risk  Blinding not possible due to the nature of the intervention | Low risk  "Independent evaluators were blinded to experimental condition throughout the study, were excluded from meetings at which condition assignment was discussed, and were not located in offices in which the CB prevention program was delivered in order to avoid inadvertent discovery of condition. In addition, at the beginning of each follow-up assessment, parents and youth were explicitly instructed not to divulge to the independent evaluator their assigned condition." (Garber et al., 2009; p.2216) | Low risk  * intention to treat design/statistical method to account for missing data: LOW RISK ("All participants were considered part of the study once randomized (i.e., an intent-to-treat design)" (Beardslee et al., 2013, p.1163))  * Difference in drop-out between groups: LOW RISK ("At the 75-month evaluation, there were no differences between those retained vs those lost to follow-up with respect to age, sex, race, ethnicity, sibling status, parent employment, adolescent depression severity or past episodes, index parent depression at baseline (IPDB), or intervention group (all *p* >.07, all *q* > .99). In the retained sample, there were no significant differences by intervention condition or site with respect to baseline variables, retention, or follow-up duration." (Brent et al., 2015, p.1112))  * Missingness related to outcome: LOW RISK ("At the 75-month evaluation, there were no differences between those retained vs those lost to follow-up with respect to age, sex, race, ethnicity, sibling status, parent employment, adolescent depression severity or past episodes, index parent depression at baseline (IPDB), or intervention group (all *p* >.07, all *q* > .99). In the retained sample, there were no significant differences by intervention condition or site with respect to baseline variables, retention, or follow-up duration." (Brent 2015, p.1112)) | Low risk  The primary outcome reported in this paper is the same as reported in the protocol. | High risk  Trial conducted by those who developed the intervention. |
| Ginsburg et al. (2009) | Unclear risk  No information specified | Unclear risk  No information specified | High risk  Blinding not possible due to the nature of the intervention | Unclear risk  They used independent evaluators, however, it is not clear whether they are naive to conditions. | Low risk  * intention to treat design/statistical method to account for missing data: LOW RISK (used intention-to-treat analysis)  * Difference in drop-out between groups: LOW RISK (dropout was similar in both groups (20 vs. 15 %))  * Missingness related to outcome: LOW RISK (Missingness is related to age and number of parents in treatment, but not to outcome) | Unclear risk  Protocol not available | High risk  Trial conducted by those who developed the intervention. |
| Ginsburg et al. (2015) | Low risk  "Eligible families were randomly assigned in a 1:1 ratio to an intervention condition (using random numbers derived from randomization. com)." (Ginsburg et al., 2015, p.1209) | Unclear risk  No information specified | High risk  Blinding not possible due to the nature of the intervention | Low risk  "All participating families were expected to complete assessments, administered by interviewers blind to intervention condition and reviewed by a senior child psychiatrist, at end of the intervention (or 8 weeks after randomization) and at followups 6 and 12 months after the 8 weeks." (Ginsburg, 2015, p.1209). | Low risk  * intention to treat design/statistical method to account for missing data: LOW RISK (used intention-to-treat analysis)  * Difference in drop-out between groups: HIGH RISK ("There were also group differences in the attrition rate, with significantly more children in the intervention condition failing to complete the 1-year assessment within the allotted window (p=0.03; see the CONSORT diagram in the online data supplement)." (Ginsburg et al., 2015; p.1210)  * Missingness related to outcome: LOW RISK (Missingness is related to age, but apparently not to other outcomes. "Attrition analysis comparing families that remained in the study and families that dropped out indicated that parents who dropped out were significantly younger on average (mean age, 41.3 years compared with 37.2 years; p=0.002). Attrition analyses for group-by-attrition interaction were not performed because of the small attrition rate (N=4) in the information-monitoring group (Ginsburg et al., 2015; p.1210)" | Low risk  The primary outcome reported in this paper is the same as reported in the protocol. | High risk  Trial conducted by those who developed the intervention. |
| Goldstein et al (2018) | Low risk  "We used a modiﬁcation of Efron's biased coin toss procedure (Efron, 1971) to randomly assigned participants to groups." (Goldstein et al., 2018; p.351) | Unclear risk  No information specified | High risk  Blinding not possible due to the nature of the intervention | Low risk  "Participants met with the study evaluator (blind to treatment condition) at intake, 3- and 6-month timepoints". The evaluator did the clinical interviews (Goldstein et al., 2018; p.351) | High risk  * intention to treat design/statistical method to account for missing data: HIGH RISK (used complete cases only; Goldstein et al., 2018; p.352)  * Difference in drop-out between groups: LOW RISK (dropout is similar in both groups (95 vs 85 %)) (Goldstein et al., 2018; p.350)  * Missingness related to outcome: UNCLEAR RISK (not reported) | Low risk   The primary outcome reported in this paper is the same as reported in the protocol. | Low risk  Trial not conducted by those who developed the intervention (not developed by Goldstein et al, but by Hlastala) |
| Mason et al (2012) | Unclear risk  No information specified | Unclear risk  No information specified | High risk  Blinding not possible due to the nature of the intervention | Unclear risk  All outcomes self-reported. Assessor blinding therefore not applicable | High risk  *intention to treat design/statistical method to account for missing data: LOW RISK ("To preserve the integrity of the randomized design, an intent-to-treat strategy was adopted in which all participants were included in the analyses based on their original random assignment to conditions rather than according to their degree of engagement in Project Hope." (Mason et al., 2012, p.899))  * Difference in drop-out between groups: HIGH RISK (Not tested specifically, but more waitlist people dropped out: "Fewer wait-list control families participated in Project Hope because they were given the option of either completing the standard in-home program or receiving a take-home version of the program for their reference, without any staff follow-up (Mason et al., 2012, p.897))."  * Missingness related to outcome: UNCLEAR RISK (not reported) | Unclear risk  Protocol not available | High risk  Trial conducted by those who developed the intervention. |
| Rasing et al (2017) | Low risk  "Directly after screening, participants were randomly allocated to the conditions, stratified on school, grade and educational level (allocation ratio (1:1)). The randomization was done by an independent researcher using a computer-generated randomization procedure." (Rasing 2017; dissertation, p.100). | Low risk  "Directly after screening, participants were randomly allocated to the conditions, stratified on school, grade and educational level (allocation ratio (1:1)). The randomization was done by an independent researcher using a computer-generated randomization procedure." (Rasing 2017; dissertation, p.100). | High risk  Blinding not possible due to the nature of the intervention | Unclear risk  All outcomes self-reported. Assessor blinding therefore not applicable | Unclear risk  * intention to treat design/statistical method to account for missing data: LOW RISK (used intention-to-treat design. Also used maximum likelihood estimation)  * Difference in drop-out between groups: UNCLEAR RISK (no test reported whether missing data differed between conditions)  * Missingness related to outcome: UNCLEAR RISK (not reported) | Low risk  The primary outcome reported in this paper is the same as reported in the protocol. | High Risk  * Trial conducted by those who developed the intervention  * One problem is that the inclusion was based on the adolescent's judgement of parental symptoms. However, the authors report later that the adolescent perception of parental problems was not very much associated with the parental self-report of psychopathology.  (Rasing, 2017; dissertation, p.144) |
| Solantaus et al. (2010) | Low risk  "The consenting families were randomized into two groups using computerized block randomization with block sizes" (Solantaus et al., 2010, p.885). | Unclear risk  No information specified | High risk  Blinding not possible due to the nature of the intervention | Unclear risk  All outcomes self-reported. Assessor blinding therefore not applicable | High risk  * intention to treat design/statistical method to account for missing data: HIGH RISK (used complete datasets and apparently not intention-to-treat analyses. "The final sample consisted of parental reports on 149 children with 83 (43 LT, 40 FTI) having complete data sets" (Solantaus et al., 2010, p.886)  * Difference in drop-out between groups: UNCLEAR RISK (not reported)  * Missingness related to outcome: LOW RISK (The drop-out analyses revealed that patient’s lower level depression (p = 0.047) and anxiety (p = 0.031) at baseline, but not children’s psychosocial symptom levels (all *p*s>0.07) predicted participation in the forthcoming data collection rounds." (Solantaus et al., 2010, p.886) | Unclear risk  Protocol not available | Low risk  Trial not conducted by those who developed the intervention (not developed by Solantaus et al, but by Beardslee). Although the control condition is developed by Solantaus et al, this is not the active intervention in this study. |
| *Note*. Risk for attrition bias was coded the following: If at least two of the three criteria were rated as low risk, then study was coded as low risk. If at least two of the three criteria were rated as unclear risk, then study was coded as unclear risk, if at least two of the three criteria were rated as high risk, then study was coded as high risk. If there were low risk, unclear risk, and high risk, then study was coded as high risk. | | | | | | | |
